# Breastfeeding duration and maternal weight change through adulthood in a population-based cohort study

**DOI:** 10.1016/j.ajcnut.2025.101134

**Published:** 2025-12-06

**Authors:** Thorbjørn B Skammelsrud, Anette Hjartåker, Sofia Klingberg, Hilde K Brekke

**Affiliations:** 1 Department of Nutrition, Institute of Basic Medical Sciences, University of Oslo, Oslo, Norway; 2 Department of Internal Medicine and Clinical Nutrition, Institute of Medicine, University of Gothenburg, Gothenburg, Sweden

**Keywords:** cohort study, epidemiology, breastfeeding, lactation, maternal health, overweight, obesity

## Abstract

**Background:**

Weight retention postpartum can increase long-term risk of maternal overweight and obesity. In theory, breastfeeding may facilitate postpartum weight loss, but its association with maternal weight change, especially long-term, remains uncertain.

**Objectives:**

The aim of this study was to investigate the association between breastfeeding duration and maternal weight change through adulthood emphasizing possible variations based on early adulthood BMI and the time of childbirth during the 1940s through the 1990s.

**Methods:**

Women (*n* = 172,472) in the Norwegian Women and Health Study, born 1927 to 1965, completed ≤3 questionnaires (Q1–Q3) between 1991 and 2014. A linear mixed model was applied to assess the association between BMI change from age 18 y in relation to mean breastfeeding duration per child (0, >0 to <3, 3 to <6, 6 to <9, 9 to <12, 12 to <15, ≥15 mo), including a 3-way interaction with categories of BMI at age 18 y and time period of first birth.

**Results:**

We found a significant interaction between breastfeeding duration per child, BMI at age 18 y, and year of first birth in relation to BMI change from age 18 y. Longer breastfeeding duration per child was associated with a lower increase in BMI among both mothers who either had overweight or obesity or had normal weight at age 18 y (*P*-trend < 0.001), irrespective of time of first birth. Among mothers with overweight or obesity at age 18 y who had their first child ≥1980, breastfeeding for ≥3 mo per child was significantly associated with lower increase in BMI from age 18 y, ranging from −1.26 kg/m^2^ [95% confidence interval (CI): −2.19, −0.32] to −2.11 kg/m^2^ (95% CI: −2.93, −1.30), compared with >0 to <3 mo.

**Conclusions:**

We found a significant association between longer breastfeeding duration per child and lower maternal weight gain through adulthood, which was particularly pronounced among mothers with overweight or obesity at age 18 y and among mothers who had their first child ≥1980.

## Introduction

Limiting weight retention after pregnancy is important for healthy long-term weight maintenance [1,2], and breastfeeding could play an important role. In theory, longer duration of breastfeeding leads to increased energy expenditure postpartum, which could result in lower weight retention, compared with shorter duration [3]. Retaining less weight, through several reproductive cycles, might lead to lower weight gain in the short and long terms. Systematic reviews provide inconclusive evidence regarding the association between breastfeeding and short-term postpartum weight retention [[4], [5], [6], [7], [8]], yet some studies and a systematic review suggest that breastfeeding promotes postpartum weight loss [[9], [10], [11]]. Furthermore, emerging evidence indicates a potential association between breastfeeding and long-term maternal weight change. A study involving >100,000 Mexican women found that mothers who breastfed >3 mo per child gained significantly less weight from age 18 y to a median of 26 y later, compared with mothers who did not breastfeed [12]. Another study of >25,000 Norwegian women found that mothers <50 y who had never lactated had >3 times the risk of obesity compared with those who had lactated for ≥24 mo in total [13].

Lifetime duration of breastfeeding is commonly used as the exposure when exploring the association between breastfeeding and long-term maternal weight change [[13], [14], [15], [16], [17]]. However, information on breastfeeding per child can provide an additional perspective and facilitate easier comparisons with established breastfeeding guidelines. Furthermore, other studies often employ “no breastfeeding” as the reference category rather than short breastfeeding duration [[12], [13], [14], [15], [16], [17], [18], [19]]. This may lead to stronger estimates, as nonbreastfeeding mothers may have distinct characteristics associated with the outcome.

Breastfeeding rates in Norway have fluctuated during the 20th century, with a considerable decline in the 1960s [[20], [21], [22]]. During the 20th century, globally, the prevalence of overweight and obesity has markedly increased among young women [23], and several studies have demonstrated that a high prepregnancy or early adulthood BMI is associated with shorter breastfeeding duration [24]. We aimed to investigate the association between breastfeeding per child and maternal weight change throughout adulthood, with particular emphasis on possible variation according to early adulthood BMI and which time during the 1940s through the 1990s the mothers gave birth to their first child.

## Methods

### Study design and study sample: the Norwegian Women and Health Study

The Norwegian Women and Health Study (NOWAC) is a large population-based prospective cohort study focused on identifying factors that influence cancer development and survival among women. The cohort and study design have been previously described in detail [[25], [26], [27]]. From 1991 to 2007, a total of 309,964 women born between 1927 and 1965 (30–70 y), randomly selected from the Norwegian Central Person Register, were invited to complete a self-administered enrollment questionnaire (Q1). Of these, 55.6% completed Q1 (*n* = 172,472). Between 1998 and 2014, 4 to 10 y after Q1, ∼70% of the first included participants (*n* ≈ 123,000) were invited to complete a second questionnaire (Q2), with 80% responding (*n* = 98,311). Subsequently, between 2004 and 2010, 4–9 y after completing Q2, roughly 63% (*n* ≈ 62,000) were invited to complete a third questionnaire (Q3), and about 79% responded (*n* = 48,953). Q2 and Q3 included many of the same questions as Q1 and were sent out to address changes in both exposures and outcomes. Nonresponders were sent a single written reminder. The reasons for nonparticipation were gathered through a small postal survey and were as follows: “too personal questions” (22.0%), “not time enough to fill in the questionnaire” (16.7%), “not interested” (6.0%), and “worried about data security in scientific studies” (28.7%). Nearly half of the nonparticipating women (49.3%) stated various other reasons; had forgotten about the questionnaire, too difficult to fill in, lost the questionnaire, language problems, and not able to fill in due to diseases [26].

The initial mailing to the first 60,000 invited participants to Q1 resulted in a low response rate among women born outside the Nordic countries. Consequently, subsequent sampling was limited to women born in Norway. Additionally, the oldest participants from Q2 were not invited to participate in Q3. The questionnaires were 2 to 8 pages long, and all of them included questions on reproductive history, hormonal factors, sociodemographic characteristics, lifestyle factors, height, and weight. Written informed consent was obtained from all participants in NOWAC, and the study has received approval from the Norwegian Data Inspectorate and the Regional Committee for Medical Research Ethics.

The current study was a secondary analysis and combined prospective and retrospective elements. To protect participants’ privacy, the dataset that was provided to us had been anonymized, and several measures had been implemented: years of education was categorized as ≤9, 10 to 12, 13 to 16, and ≥17 y; age at first birth was categorized as <20, 20 to 24, 25 to 29, and ≥30 y; age at last birth was categorized as <20, 20 to 24, 25 to 29, 30 to 34, 35 to 39, and ≥40 y; women with a calculated BMI of <15 kg/m^2^ or >35 kg/m^2^ at age 18 y (0.7%) were assigned values of 15 or 35 kg/m^2^, respectively; women with a calculated current BMI <15 kg/m^2^ or >50 kg/m^2^ at the time of Q1 to Q3 (0.1%) were assigned values of 15 or 50 kg/m^2^, respectively; parity and breastfeeding data on women with ≥6 children (0.9%) were replaced with missing values (and thus excluded from data analysis). Months of breastfeeding per child were retrospectively reported in Q1 without distinguishing between exclusive and partial breastfeeding. We converted mean breastfeeding duration per child from a continuous to a categorical scale (0, 0 to <3, 3 to <6, 6 to <9, 9 to <12, 12 to <15, ≥15 mo) to facilitate comparison between breastfeeding durations. Current age (continuous), current height and weight (continuous), current smoking status (never, former, current), and current physical activity level were reported in Q1, Q2, and Q3. Physical activity at work, household chores, walking, and organized exercise from a 10-point scale were categorized as: “low” = 1 to 4, “medium” = 5 to 6, and “high” = 7 to 10. The 10-point scale has previously been validated in a female Norwegian population [28]. Estimated year of first birth was calculated by adding the birth year of each mother to a value we selected in their respective “age at first birth” category. The values were 18, 22, 27, and 32 in the “age at first birth” categories, respectively. BMI at age 18 y and at the time of Q1, Q2, and Q3 was calculated from reported height and weight. Weight at age 18 y was retrospectively collected in Q1. The outcomes, BMI change from age 18 y to the time of Q1, Q2, and Q3, were calculated by subtracting BMI at age 18 y from the current BMI at Q1, Q2, and Q3, respectively.

### Participant flow

**FIGURE 1:**
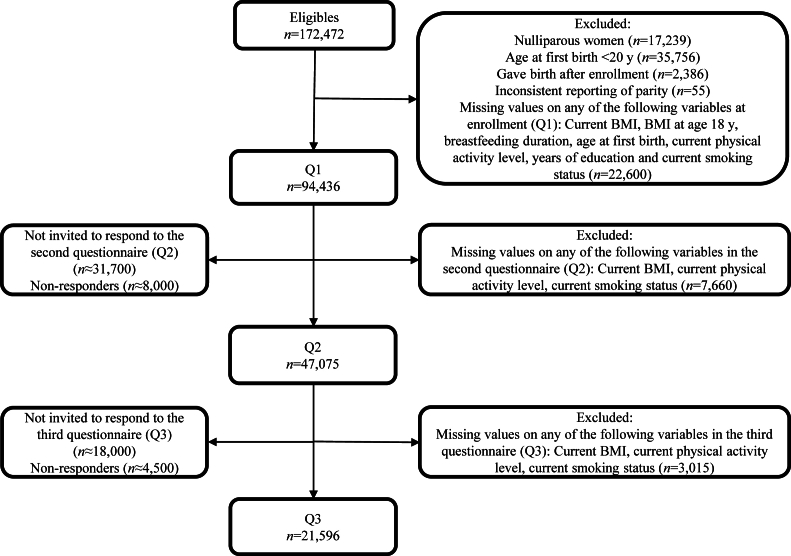
Flowchart showing the selection of participants in the current study from NOWAC. NOWAC, The Norwegian Women and Health Study; Q1, enrollment/first questionnaire; Q2, second questionnaire; Q3, third questionnaire.

In the current study, all women with missing data on relevant variables at Q1 (*n* = 22,600), Q2 (*n* = 7660), and Q3 (*n* = 3015) were excluded (Figure 1). Nulliparous women were excluded (*n* = 17,239). Additionally, to ensure that weight at age 18 y was before the mothers’ first pregnancies, we excluded all women who had given birth before the age of 20 y (*n* = 35,756). We further excluded those who gave birth after enrollment (*n* = 2386) and the women who had inconsistent reporting of parity (*n* = 55).

### Statistical analyses

The descriptive characteristics of the total NOWAC cohort and the participants are presented as percentages, mean with SD or min–max for normally distributed variables, and median values with 25th and 75th percentiles or min–max for non-normally distributed variables. The association between breastfeeding duration per child and change in BMI from age 18 y was examined using linear mixed models with >0 to <3 mo per child as the reference category. The linear mixed model analysis included participants with missing data for the dependent variable. We assessed multicollinearity by conducting a variance inflation factor test and included dummy variables for all categorical variables. On the basis of existing literature and prior knowledge, current age, current smoking status, current physical activity level, years of education, age at first birth and parity were used as fixed effects. Directed acyclic graphs were employed to determine the factors that should be adjusted for, based on the assumed associations (Supplemental Figures 1 and 2). A random intercept for participant number was included to account for within-subject correlation. BMI at age 18 y (underweight: <18.5 kg/m^2^; normal weight: 18.5 to 24.9 kg/m^2^; overweight or obesity: ≥25 kg/m^2^) and estimated year of first birth (<1970, 1970 to 1979, ≥1980) were included in a 3-way interaction with breastfeeding duration per child. Stratum-specific estimates were calculated for each combination of “BMI at age 18” category and birth-year cohort. All statistical analyses were conducted using STATA SE version 18.0, and statistical tests used a 2-tailed alpha of 0.05.

## Results

Most of the women had normal weight at age 18 y (79%), had their first child between 1970 and 1979 (53%), and reported giving birth to 2 children (50%) in Q1 (Table 1). We estimated that mothers had their last parturition, and thus their final breastfeeding period, at a mean of 21 y (range: 0–50) before Q1, 24 y (range: 2–54) before Q2, and 26 y (range: 6–49) before Q3. The mean difference in BMI from age 18 y to the respective questionnaires was 3.3 kg/m^2^ (SD: 3.7), 3.9 kg/m^2^ (SD: 3.8), and 4.4 kg/m^2^ (SD: 3.9). Current smokers varied from 27% (Q1) to 21% (Q3) and the majority reported a medium physical activity level (Table 2). To illustrate the variation in characteristics, we present data stratified by categories of mean breastfeeding duration per child (Table 3). Mean current age among those who had their first child before 1970, 1970 to 1979 and from 1980 and later were 55.7 (SD: 7.0) y, 48.1 (SD: 7.2) y, and 44.6 (SD: 7.1) y at Q1, respectively.

**TABLE 1:** Characteristics of NOWAC and the study participants at Q1, Q2, and Q3

Characteristic	NOWAC^1^*n* = 172,472	Q1 *n* = 94,436	Q2 *n* = 47,075	Q3 *n* = 21,596
Breastfeeding duration per child^2^ (mo)	4.7 (0, 72)	5.5 (0, 72)	5.3 (0, 66)	5.3 (0, 48)
BMI^3^ (kg/m^2^) at age 18 y	20.8 (2.6)	20.8 (2.5)	20.8 (2.5)	20.7 (2.4)
BMI (kg/m^2^) at age 18 y %				
<18.5	16.1	15.8	14.8	14.9
18.5–24.9	78.4	79.3	80.3	80.7
≥25	5.5	4.9	4.9	4.5
Estimated year of first birth %				
1947–1969	37.5	27.9	30.0	22.5
1970–1979	47.8	52.9	52.5	60.9
1980–1997	14.7	19.2	17.5	16.6
Parity %				
0	10.0	—	—	—
1	12.0	14.6	13.9	13.8
2	41.5	49.7	50.1	52.6
3	25.6	27.0	27.5	26.9
4	7.9	7.1	7.0	5.6
5	2.1	1.6	1.5	1.1
≥6	0.9	—	—	—
Age at first birth (y; %)				
<20	23.0	—	—	—
20–24	45.9	59.3	60.1	60.9
25–29	22.6	29.7	30.1	30.0
≥30	8.5	11.0	9.8	9.1
Education (y; %)				
<10	23.1	16.3	15.8	13.8
10–12	34.3	33.3	34.0	35.0
13–16	27.7	32.6	33.8	35.6
≥17	8.5	17.8	16.4	15.6

All data collected retrospectively at Q1.

Abbreviations: NOWAC, The Norwegian Women and Health Study; Q1, enrollment/first questionnaire; Q2, second questionnaire; Q3, third questionnaire.

1 Data for some participants were missing.

2 Presented as median (min, max).

3 Presented as mean (SD).

**TABLE 2:** Characteristics of NOWAC and the study participants at Q1, Q2, and Q3

Characteristic	NOWAC 1*n* = 172,472	Q1 *n =* 94,436	Q2 *n =* 47,075	Q3 *n* = 21,596
Current age (y) 2	49.5 (31, 70)	49.5 (31, 70)	53.3 (35, 76)	55.7 (39, 68)
Current BMI (kg/m^2^) 3	24.3 (3.9)	24.1 (3.8)	24.7 (3.8)	25.2 (4.0)
Current smoking status (%)				
Never	34.7	37.2	42.0	40.0
Former	34.5	36.3	34.9	39.5
Current	30.8	26.5	23.1	20.5
Current physical activity level (%)				
Low (1–3)	24.8	23.7	26.1	20.1
Medium (4–6)	41.2	41.9	43.5	41.3
High (7–10)	34.0	34.4	30.4	38.6

All data collected prospectively.

Abbreviations: NOWAC, The Norwegian Women and Health Study; Q1, enrollment/first questionnaire; Q2, second questionnaire; Q3, third questionnaire.

1 Data for some participants were missing.

2 Presented as mean (min, max).

3 Presented as mean (SD).

**TABLE 3:** Characteristics of study participants at Q1 by categories of mean breastfeeding duration per child

Characteristic	Mean breastfeeding duration per child (mo) *n =* 94,436						
	0 *n =* 6289 (6.7%)	>0 to <3 *n =* 16,931 (17.9%)	3 to <6 *n =* 25,949 (27.5%)	6 to <9 *n =* 21,340 (22,6%)	9 to <12 *n =* 15,053 (15.9%)	12 to <15 *n =* 6024 (6.4%)	≥15 *n =* 2850 (3.0%)
Breastfeeding duration per child^1^ (mo)	0	1.7 (1, 2)	4.0 (3, 5)	7.0 (6, 8)	10.0 (9, 11)	12.3 (12, 13)	18.0 (16, 22)
Current age^2^ (y)	50.7 (8.2)	50.4 (8.1)	50.2 (8.3)	49.0 (8.4)	48.4 (8.0)	48.3 (7.3)	48.1 (7.0)
Estimated year of first birth %							
1947–1969	38.8	42.7	35.4	22.6	13.6	8.2	5.4
1970–1979	47.2	48.7	52.2	56.8	56.6	52.4	49.2
1980–1997	14.0	8.6	12.4	20.6	29.8	39.5	45.4
Current BMI 2 (kg/m^2^)	25.0 (4.5)	24.4 (4.0)	24.2 (2.8)	23.9 (2.4)	23.7 (3.6)	23.7 (3.6)	24.0 (3.9)
BMI (kg/m^2^) at age 18 y 2	21.2 (3.0)	20.9 (2.7)	20.8 (2.5)	20.7 (2.4)	20.7 (2.3)	20.6 (2.4)	20.5 (2.3)
BMI (kg/m^2^) at age 18 y (%)							
<18.5	15.0	15.7	15.9	15.9	15.3	16.5	17.2
18.5–24.9	76.3	77.9	79.3	80.3	80.9	79.4	78.5
≥25	8.7	6.4	4.8	3.8	3.8	4.1	4.3
Parity^2^ (*n*)	2.0 (0.9)	2.3 (0.9)	2.3 (0.9)	2.4 (0.8)	2.3 (0.9)	2.2 (0.9)	2.1 (0.9)
Current physical activity level^2^	5.5 (2.0)	5.5 (1.9)	5.7 (1.8)	5.8 (1.8)	5.9 (1.8)	5.9 (1.8)	5.9 (1.9)
>17 y of education %	9.2	8.4	12.1	19.3	28.5	35.7	39.7
>30 y of age at first birth %	13.0	6.0	6.9	9.8	15.0	23.9	33.1
Current smokers %	32.3	34.9	30.2	22.8	18.5	17.4	19.7

Abbreviation: Q1, enrollment/first questionnaire.

1 Presented as median (25th and 75th percentiles)

2 Presented as mean (SD).

**FIGURE 2:**
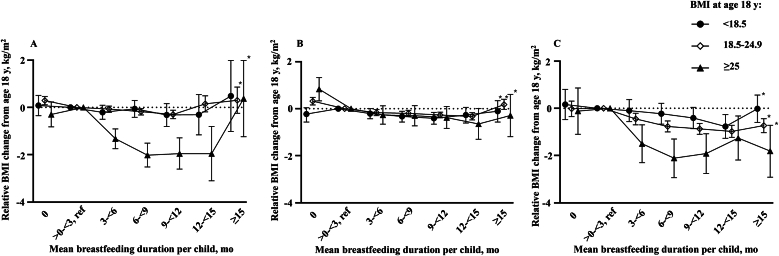
Associations between mean duration of breastfeeding per child and maternal BMI change from age 18 y to midlife among mothers who had their first birth in the 1940s to 1960s (A) (*n* = 27,676), 1970s (B) (*n* = 51,904), and 1980s to 1990s (C) (*n* = 18,623). The shown estimates and 95% CIs were estimated by linear mixed model analyses with a 3-way interaction term between breastfeeding duration per child, categories of BMI at age 18 y, and categories of year of first birth. Adjusted for current age, current physical activity level, current smoking status, parity, years of education, and age at first birth. The error bars represent the 95% CIs. Reference line: 0 kg/m^2^. ∗*P*-trend < 0.05. CI, confidence interval.

The linear mixed-effects model demonstrated a significant 3-way interaction among breastfeeding duration per child, BMI at age 18 y, and year of first birth (*P* < 0.001), after adjusting for current age, parity, years of education, age at first birth, current smoking status, and current physical activity level (Figure 2A–C). This shows that the effect modification by categories of BMI at age 18 y, in the association between breastfeeding duration per child and BMI change from age 18 y, varies significantly across categories of year of first birth.

In all 3 categories of year of first birth, longer breastfeeding duration per child was associated with a lower increase in BMI among both mothers who either had overweight or obesity or had normal weight at age 18 y (*P*-trend < 0.001) (Figure 2A–C). This trend was also evident among mothers with underweight at age 18 y giving birth to their first child in the 1970s or after 1979 (*P*-trend < 0.05) (Figure 2B, C).

### First child born before 1970

Among those who had overweight or obesity at age 18 y, mothers who breastfed for 3 to <6, 6 to <9, 9 to <12, and 12 to <15 mo per child had lower increase in BMI than mothers who breastfed for >0 to <3 mo (−1.32 to −2.02 kg/m^2^, *P* < 0.001) (Figure 2A and Supplemental Table 1). Mothers who had normal weight at age 18 y, who did not breastfeed, had a significantly higher increase in BMI than mothers who breastfed for >0 to <3 mo per child, 0.29 kg/m^2^ [95% confidence interval (CI): 0.11, 0.46]. In contrast, mothers with normal weight at age 18 y who breastfed 6 to <9 mo and 9 to <12 mo, had a lower increase in BMI than the reference category (−0.18 to −0.30 kg/m^2^, *P* < 0.001). We found no significant associations among women who had underweight at age 18 y and had their first child before 1970.

### First child born from 1970 to 1979

Both mothers with overweight or obesity and those with normal weight at age 18 y, who did not breastfeed, had a significantly higher increase in BMI than mothers who breastfed for >0 to <3 mo per child, 0.83 kg/m^2^ (95% CI: 0.33, 1.32) and 0.32 kg/m^2^ (95% CI: 0.16, 0.48), respectively (Figure 2B and Supplemental Table 1). Additionally, mothers with normal weight at age 18 y who breastfed for 3 to <6, 6 to <9, 9 to <12, and 12 to <15 mo per child, had a significantly lower increase in BMI than the reference category (−0.17 to −0.33 kg/m^2^, *P* < 0.001). Mothers with underweight at age 18 y, who breastfed for 6 to <9 and 9 to <12 mo per child, had a significantly lower increase in BMI than mothers who breastfed for >0 to <3 mo (−0.33 to −0.41 kg/m^2^, *P* < 0.001).

### First child born after 1979

Mothers with overweight or obesity at age 18 y, who breastfed for 3 to <6, 6 to <9, 9 to <12, 12 to <15 and ≥15 mo per child had a significantly lower increase in BMI than the reference category (−1.26 to −2.11 kg/m^2^, *P* < 0.001) (Figure 2C and Supplemental Table 1). Among mothers with normal weight, breastfeeding for 3 to <6, 6 to <9, 9 to <12, 12 to <15, and ≥15 mo per child was significantly associated with a lower increase in BMI (−0.45 to −0.98 kg/m^2^, *P* < 0.001). We also found that mothers with underweight at age 18 y who breastfed for 12 to <15 mo per child had a significantly lower increase in BMI than mothers who breastfed for >0 to <3 mo (−0.77 kg/m^2^ 95% CI: −0.27, −1.27).

## Discussion

In this large Norwegian cohort, longer mean breastfeeding duration per child was associated with lower weight gain from age 18 y among mothers with overweight or obesity and normal weight at age 18 y across all categories of year of first birth. We observed a significant effect modification by BMI at age 18 y and year of first birth based on a 3-way interaction with breastfeeding duration per child. The association between breastfeeding duration and weight change from age 18 y was particularly pronounced among mothers who had their first child in the 1980s or 1990s and among those who had overweight or obesity at age 18 y. Among mothers with normal weight at age 18 y, the benefit of breastfeeding became increasingly apparent as the mother’s first birth occurred later in the 20th century. There was some support of an association between longer breastfeeding duration and lower maternal weight gain also among those with underweight at age 18 y.

In our study, mothers with overweight or obesity at age 18 y who breastfed for 6 to <9 mo gained ≤6.5 kg (2.11 kg/m^2^) less than those who breastfed for >0 to <3 mo per child, whereas mothers with normal weight at age 18 y gained ≤3.0 kg (0.98 kg/m^2^) less. These estimates exceed those in previous studies, although the results are not directly comparable. A British study of >740,000 mothers, with a mean age of 57.5 y, showed that those who had a lifetime duration of breastfeeding of ≥10 mo had a BMI that was 0.53 kg/m^2^ lower compared with mothers who did not breastfeed at all [18]. A study from Mexico with >100,000 mothers indicated that those who breastfed 3 to 6 mo per child gained 1.1 kg less, from age 18 to 44 y, than mothers who did not breastfeed [12]. This Mexican study is among the few to investigate the interaction between prepregnancy BMI or early adulthood BMI and breastfeeding duration, but no significant interaction was found. Contradicting our findings, another study reported that mothers with prepregnancy obesity gained more weight when breastfeeding for longer [29]. Differences between results may be explained by differences in methods and population characteristics.

Among mothers with overweight or obesity at age 18 y, the association pattern in our study was distinct for those whose first birth occurred in the 1970s, as no breastfeeding was associated with a significantly greater weight gain than breastfeeding for >0 to <3 mo, whereas breastfeeding beyond 3 mo was not associated with lower weight gain (Figure 2B). Increased formula uses in the 1970s could have shortened the period of exclusive breastfeeding [30], diminishing the energy expenditure from breastfeeding that might have reduced long-term weight gain.

The association between breastfeeding and weight change became more apparent among those with normal weight at age 18 y, as the mothers had their first child later in the 20th century. From the 1960s through the 1990s, the benefits of breastfeeding became more widely known. For example, in 1968, the organization “Ammehjelpen,” comprising experienced breastfeeding women and midwives, was established in Norway to provide advice to breastfeeding mothers. Such factors contributed to increasing breastfeeding rates [22], which is also evident in NOWAC (data not shown). Mothers who gave birth to their first child before 1970 also had the highest mean age at enrollment in our study, suggesting that the differing associations could be influenced by their older age and the menopausal transition. A Norwegian study showed a significant association between longer breastfeeding duration and lower prevalence of obesity among mothers <50 y but not among women aged ≥50 y [13]. However, in a Polish–Norwegian study, longer breastfeeding was associated with lower risk of excessive weight among postmenopausal but not premenopausal women [14]. Conflicting findings may be related to differing time period of childbirth as much as the age of the women at adiposity measurement.

Some limitations in our study must be acknowledged. Our analysis was restricted to evaluating any breastfeeding duration, and thus, we could not assess breastfeeding exclusivity or intensity. We chose to use the available data on breastfeeding per child, as it provides insights for practical application. This limits our ability to compare with studies that used lifetime breastfeeding duration. However, also in our cohort, lifetime duration of breastfeeding (>0 to <7, 7 to <12, 12 to <24, ≥24 mo) was associated with lower increase in BMI, compared with no breastfeeding (data not shown). The mothers reported breastfeeding duration and weight at age 18 y retrospectively, in some cases ≤50 y after, which may have introduced recall errors. However, several studies have demonstrated moderate to strong agreement between recalled and prospectively collected data on breastfeeding duration [31,32] and weight [[33], [34], [35]], even at older ages. For example, a weighted kappa statistic of 0.55 was reported when comparing recalled and prospectively collected breastfeeding duration in women aged 69 to 79 y [32]. Additionally, self-reported weight and height provide a valid ranking of BMI for middle-aged women in NOWAC [36]. Weight is commonly underestimated when self-reported, especially among women with overweight or obesity [36,37]. This could have led to biased estimates as women with higher BMI also have shorter breastfeeding duration [24]. Furthermore, BMI at 18 y had to be used as a proxy for prepregnancy BMI. Weight gain from age 18 y to first pregnancy may be associated with both shorter breastfeeding duration and higher weight in midlife. Hence, using BMI at age 18 y as proxy may have resulted in inflated estimates. However, adjusting for age at first birth, and thus the time for weight change to occur, could have reduced such bias. Residual confounding may persist, especially as behavioral factors, like smoking and physical activity, were restricted to midlife. Although not confounders by definition in this analysis, we included current smoking status and physical activity level in the model due to their relevance as predictors of current BMI.

A strength of our study is the high response rate, which reduces potential selection bias and enhances generalizability. Also, NOWAC is broadly representative of the source population, aside from slightly higher educational levels and few participants born outside the Nordic countries [26]. However, we cannot dismiss the potential influence of loss to follow-up on the current results. Multiple weight recordings during midlife and extensive breastfeeding data, reflecting trends spanning several decades, allowed for a thorough examination of breastfeeding’s long-term association with weight change. Importantly, we limited the risk of reverse causality by using BMI change from age 18 y as the outcome while only including women who had children after age 20 y. Unlike many prior studies, we selected short breastfeeding duration per child (>0 to <3 mo) as the reference rather than no breastfeeding, as nonbreastfeeding mothers may differ systematically in characteristics related to the outcome. This approach likely produced more conservative estimates and strengthened the validity of our findings.

In conclusion, our study expands on the existing literature as we demonstrated an association between breastfeeding duration per child and long-term weight change in a large Norwegian cohort. We found a significant 3-way interaction with BMI at age 18 y and year of first birth, indicating the importance of these factors for the current association. Breastfeeding >3 mo per child was especially beneficial among mothers with overweight or obesity at age 18 y having their first child after 1980, making the findings highly relevant for women today. Our findings suggest that breastfeeding duration may play an important role in the prevention of maternal weight gain through childbearing years and beyond.

## Author contributions

The authors’ responsibilities were as follows – HKB, AH: designed the research; TBS: analyzed data, wrote the paper, and had primary responsibility for the final content; SK, AH, HKB: contributed to the review and editing of the manuscript; and all authors: read and approved the final manuscript.

## Data availability

Data described in the manuscript, code book, and analytic code will be made available upon reasonable request to nowac@uit.no by following the steps presented on the website https://uit.no/research/nowac_en#region_783025.

## Declaration of generative AI and AI-assisted technologies in the writing process

During the preparation of this work, the authors used “GPT UiO” to assist with language editing and enhancing readability. After using this tool/service, the authors reviewed and edited the content as needed and take full responsibility for the content of the publication.

## Funding

This work was supported by the Throne-Holst Foundation for Nutrition Research. The funder did not have any role in the design, execution or interpretation of the research; or had any restrictions regarding publication.

## Conflict of interest

The authors report no conflicts of interest.
