# Supplementary material for: Breastfeeding duration and maternal weight change through adulthood in a population-based cohort study
Source: Am J Clin Nutr. 2025 Dec 6;123(2):101134. doi: 10.1016/j.ajcnut.2025.101134 (PMC12917222; doi:10.1016/j.ajcnut.2025.101134)

**Supplementary Material**

| **Supplementary Table 1** Stratum-specific estimates of the association between mean breastfeeding duration per child and maternal BMI change from age 18 y, by categories of BMI at age 18 y and categories of year of first birth (model include an interaction term: breastfeeding duration per child × BMI at age y × year of first birth)^1^. | | |
| --- | --- | --- |
|  | Adjusted BMI change, kg/m^2^ ^2^ | p trend |
| Breastfeeding per child, mo  Year of first birth: <1970  BMI at age 18 y: <18.5 kg/m^2^  (*n*=5,719) ^3^ |  | 0.20 |
| 0 | 0.08 (-0.35, 0.51) |  |
| >0-<3 (reference) | 0 |  |
| 3-<6 | -0.21 (-0.50, 0.09) |  |
| 6-<9 | -0.06 (-0.42, 0.29) |  |
| 9-<12 | -0.32 (-0.81, 0.16) |  |
| 12-<15 | -0.31 (-1.16, 0.54) |  |
| ≥15 | 0.48 (-1.02, 1.98) |  |
| BMI at age 18 y: 18.5-24.9 kg/m^2^  (*n*=38,959) ^3^ |  | <0.001 |
| 0 | 0.29 (0.11, 0.46) |  |
| >0-<3 (reference) | 0 |  |
| 3-<6 | -0.07 (-0.21, 0.06) |  |
| 6-<9 | -0.18 (-0.32, -0.05) |  |
| 9-<12 | -0.30 (-0.48, -0.12) |  |
| 12-<15 | 0.15 (-0.19, 0.49) |  |
| ≥15 | 0.30 (-0.25, 0.86) |  |
| BMI at age 18 y: ≥25 kg/m^2^  (*n*=2,839) ^3^ |  | <0.001 |
| 0 | -0.30 (-0.82, 0.23) |  |
| >0-<3 (reference) | 0 |  |
| 3-<6 | -1.32 (-1.74, -0.91) |  |
| 6-<9 | -2.02 (-2.52, -1.51) |  |
| 9-<12 | -1.95 (-2.61, -1.29) |  |
| 12-<15 | -1.95 (-3.10, -0.81) |  |
| ≥15 | 0.37 (-1.24, 1.98) |  |
| Year of first birth: 1970-1979  BMI at age 18 y: <18.5 kg/m^2^  (*n*=15,059) ^3^ |  | 0.03 |
| 0 | -0.23 (-0.57, 0.04) |  |
| >0-<3 (reference) | 0 |  |
| 3-<6 | -0.21 (-0.43, 0.00) |  |
| 6-<9 | -0.33 (-0.57, -0.11) |  |
| 9-<12 | -0.41 (-0.66, -0.16) |  |
| 12-<15 | -0.27 (-0.61, 0.06) |  |
| ≥15 | -0.11 (-0.57, 0.34) |  |
| BMI at age 18 y: 18.5-24.9 kg/m^2^  (*n*=72,739) ^3^ |  | <0.001 |
| 0 | 0.32 (0.16, 0.48) |  |
| >0-<3 (reference) | 0 |  |
| 3-<6 | -0.17 (-0.30, -0.04) |  |
| 6-<9 | -0.20 (-0.33, -0.07) |  |
| 9-<12 | -0.27 (-0.39, -0.15) |  |
| 12-<15 | -0.33 (-0.48, -0.17) |  |
| ≥15 | 0.18 (-0.03, 0.40) |  |
| BMI at age 18 y: ≥25 kg/m^2^  (*n*=4,194) ^3^ |  | <0.001 |
| 0 | 0.83 (0.33, 1.32) |  |
| >0-<3 (reference) | 0 |  |
| 3-<6 | -0.27 (-0.66, 0.12) |  |
| 6-<9 | -0.30 (-0.73, 0.13) |  |
| 9-<12 | -0.38 (-0.84, 0.09) |  |
| 12-<15 | -0.65 (-1.30, 0.01) |  |
| ≥15 | -0.29 (-1.19, 0.61) |  |
| Year of first birth: ≥1980  BMI at age 18 y: <18.5 kg/m^2^  (*n*=5,434) ^3^ |  | <0.01 |
| 0 | 0.16 (-0.48, 0.80) |  |
| >0-<3 (reference) | 0 |  |
| 3-<6 | -0.10 (-0.56, 0.37) |  |
| 6-<9 | -0.23 (-0.67, 0.21) |  |
| 9-<12 | -0.41 (-0.86, 0.04) |  |
| 12-<15 | -0.77 (-1.27, -0.27) |  |
| ≥15 | -0.02 (-0.60, 0.56) |  |
| BMI at age 18 y: 18.5-24.9 kg/m^2^  (*n*=24,137) ^3^ |  | <0.001 |
| 0 | -0.02 (-0.35, 0.31) |  |
| >0-<3 (reference) | 0 |  |
| 3-<6 | -0.45 (-0.70, -0.21) |  |
| 6-<9 | -0.77 (-1.00, -0.53) |  |
| 9-<12 | -0.87 (-1.10, -0.64) |  |
| 12-<15 | -0.98 (-1.23, -0.72) |  |
| ≥15 | -0.73 (-1.03, -0.44) |  |
| BMI at age 18 y: ≥25 kg/m^2^  (*n*=1,374) ^3^ |  | <0.001 |
| 0 | -0.12 (-1.10, 0.86) |  |
| >0-<3 (reference) | 0 |  |
| 3-<6 | -1.50 (-2.30, -0.70) |  |
| 6-<9 | -2.11 (-2.93, -1.30) |  |
| 9-<12 | -1.91 (-2.76, -1.08) |  |
| 12-<15 | -1.26 (-2.19, -0.32) |  |
| ≥15 | -1.81 (-2.91, -0.72) |  |
| ^1^ Estimates and 95% CIs were estimated by linear mixed model analyses.  ^2^ Adjusted for current age, parity, current physical activity level, current smoking status,  years of education, and age at first birth.  ^3^ Mixed model observations. | | |

**Supplementary Figure 1.** Conceptual framework, unadjusted (pink arrows indicate bias).


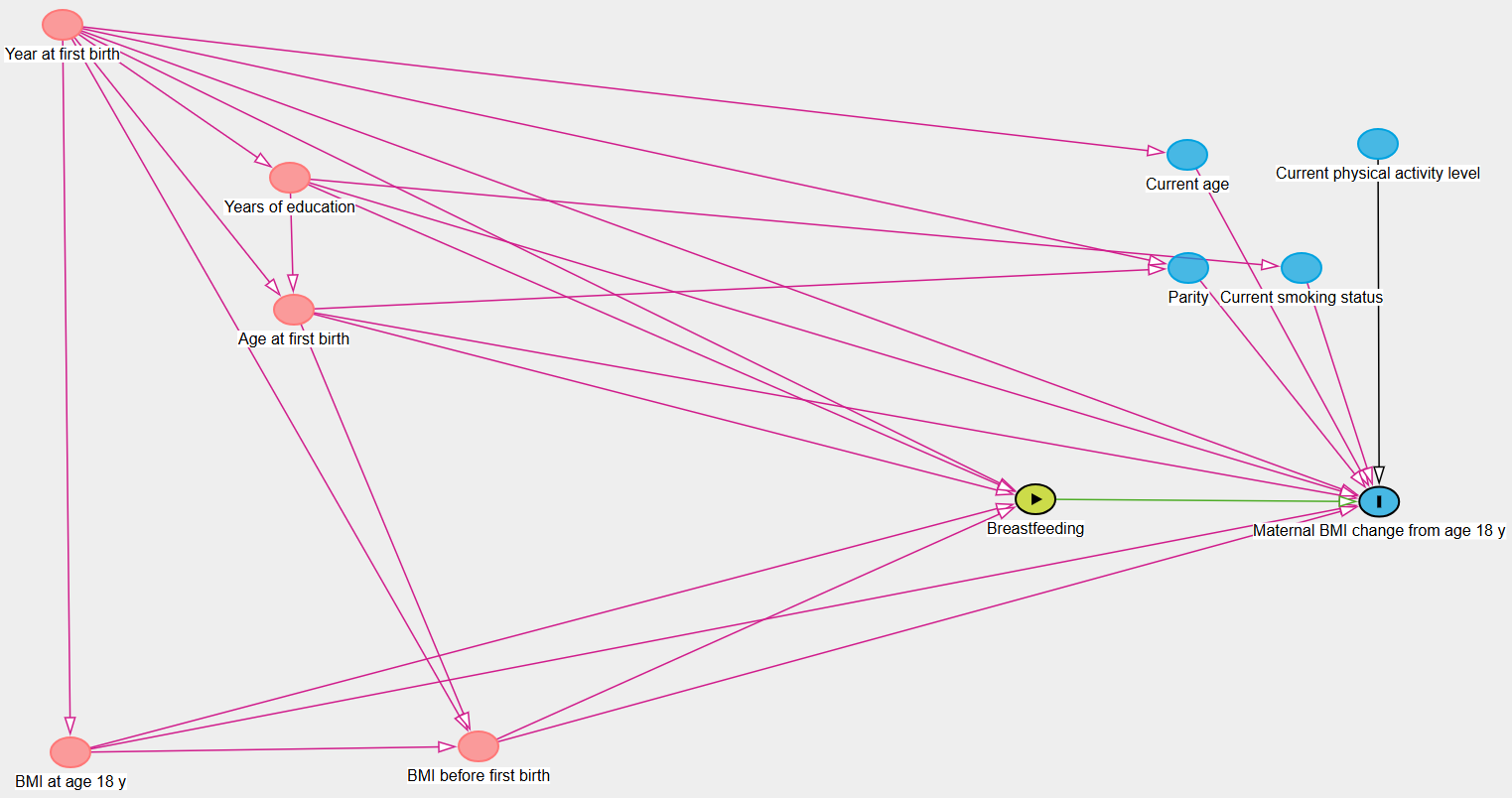


**Supplementary Figure 2.** Conceptual framework, adjusted. Adjusted for current age, current smoking status, current physical activity level, years of education, age at first birth and parity. Stratified by year at first birth and BMI at age 18 y.


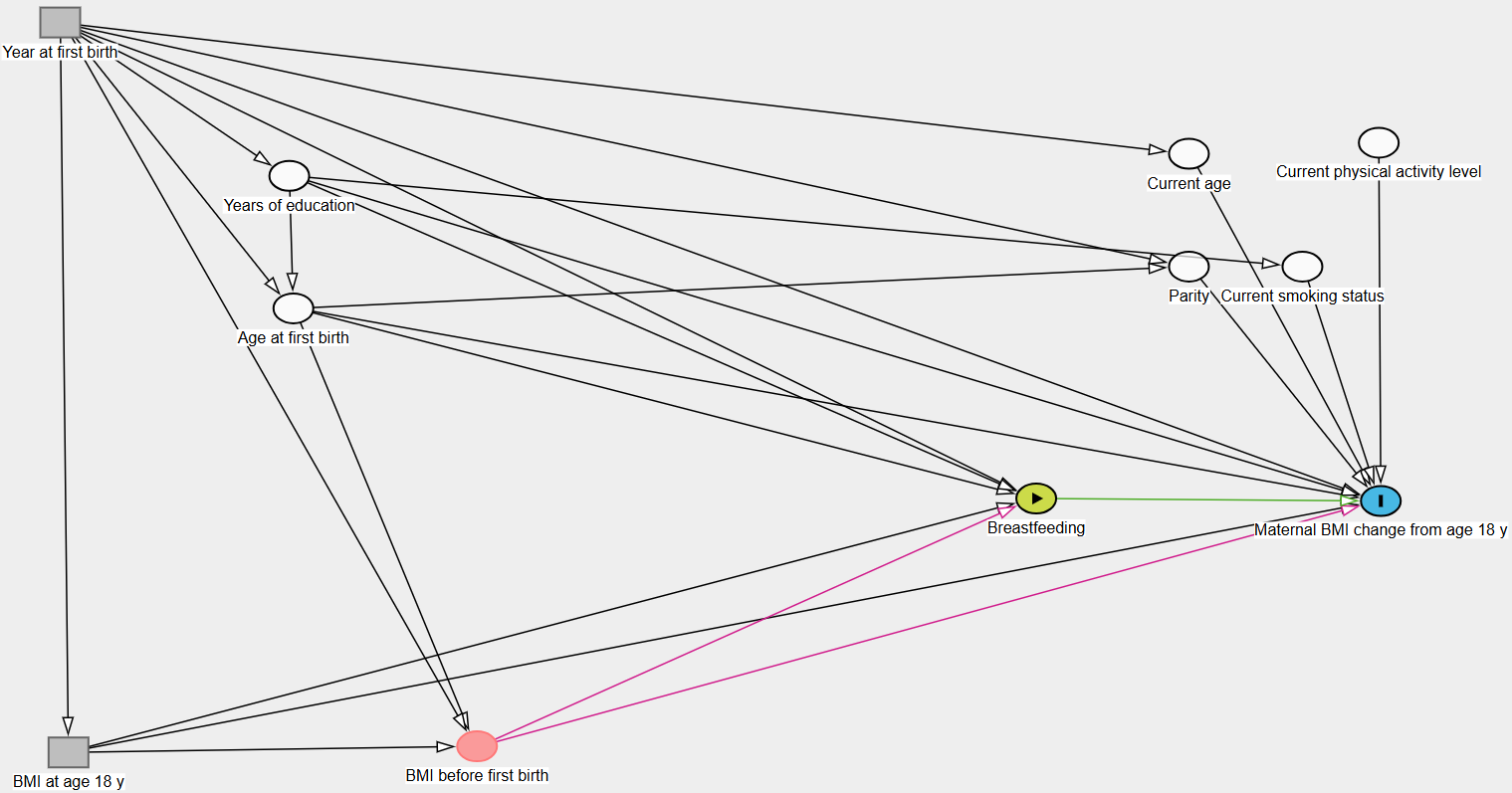

Supplement: Multimedia component 1 [file mmc1.docx]
